# Knowledge, beliefs and practices of nurses with long-acting reversible contraception, Cape Town

**DOI:** 10.4102/phcfm.v15i1.3571

**Published:** 2023-05-24

**Authors:** Tracey-Leigh Abrahams, Michael K. Pather, Steve Swartz

**Affiliations:** 1 Department of Family and Emergency Medicine, Faculty of Medicine and Health Sciences, Stellenbosch University, Cape Town, South Africa

**Keywords:** long-acting reversible contraception, primary health care, intrauterine contraceptive device, Implanon, intrauterine contraceptive device

## Abstract

**Background:**

Implanon and copper intrauterine contraceptive device (IUCD) are long-acting reversible contraceptives (LARC) available in public primary health care (PHC) South Africa. These methods are the most effective forms of contraception.

**Aim:**

To evaluate the knowledge, beliefs and practices on provision of LARC.

**Setting:**

Primary health care facilities within the Khayelitsha Eastern Substructure, Cape Town.

**Methods:**

A descriptive survey of all permanent nurses who provided contraception. Data were collected from 72/90 (80% response rate) via a validated questionnaire and evaluated using Statistical Package for Social Sciences (SPSS).

**Results:**

Knowledge of eligibility for LARC was tested. The mean knowledge scores for Implanon were 8.56/11 (s.d. 1.42) for the trained and 7.16/11 (s.d. 2.83) for the untrained (*p* = 0.007). The mean knowledge scores for IUCD were 10.42/12 (s.d. 1.80) for the trained and 8.03/12 (s.d. 3.70) for the untrained (*p* = 0.019). Participants believed that inaccessibility to training courses (29%), no skilled person available (24%) and staff shortages (35%) were barriers. Less than 50% of women were routinely counselled for LARC. Forty-one percent of nurses were trained and performed IUCD insertion, and 64% were trained and performed Implanon insertion, while 61% and 45% required further training. Confidence was low, with 32% trained and confident in IUCD and 56% trained and confident in Implanon insertion.

**Conclusion:**

Lack of training, poor confidence and deficient counselling skills were barriers to effective LARC provision. The identified system-specific barriers must be addressed to improve uptake.

**Contribution:**

The first study to evaluate knowledge, beliefs and practices on LARC in providers in the Western Cape.

## Introduction

Long-acting reversible contraceptives (LARC) are highly effective, but are currently underutilised worldwide.^1,2,3,4,5,6^ Globally, subdermal implants are underused despite their excellent efficacy, and worldwide, fewer than 1% of women in their reproductive age use implants for contraception.^7^ Countries including the United States, Canada, England and Australia report that less than 10% of reproductive-age women are using an intrauterine contraceptive device (IUCD).^7^ In South Africa, short-acting contraceptives such as condoms, injectables and oral contraception take preference as the choice of contraception among women of childbearing age.^1,8,9^ The Implanon and the copper IUCD are the two forms of LARC that are available in the primary health care (PHC) setting in South Africa.

A 2012 worldwide study showed that of the 213 million pregnancies that occurred, 85 million pregnancies, representing 40%, were unintended.^10^ Of these, 50% ended in abortion, 13% ended in miscarriage and 38% resulted in an unplanned birth.^10^ Worldwide, unintended pregnancies are a public health concern that account for substantial costs to healthcare systems.^11^ Research conducted in Brazil claimed that part of the high rates of unplanned pregnancies may be attributed to the relatively low use of LARC methods.^12^ In Nigeria, illegal abortions arising from unwanted pregnancies constitute a major reproductive health problem, and data indicate that the proportion of women having unwanted pregnancies in this country is increasing.^13^ Data from a South African household survey in 2012 assessed contraception coverage among women aged 15–49 years. The study concluded that two-thirds of women had an unintended pregnancy in the 5 years preceding the study, a quarter of which were as a result of contraceptive failures.^1^

Long-acting reversible contraceptive methods are proven to be the most effective form of contraception, cost-effective and suitable for a wide variety of women.^14,15,16,17,18^ With typical use, the failure rate of the IUCD is 1% and that of the Implanon is 0.1% in the 1st year of use.^19^ One-year continuation rates are also markedly superior to short-acting reversible contraceptives, such as the progesterone-only pills, injectables and the combined oral contraceptives.^19^

Previously conducted international studies have shown that poor counselling from contraception providers, lack of training and poor provider knowledge, lack of skill and certain beliefs of the provider can act as barriers in terms of the provision of LARC.^2,20,21,22,23^ Research conducted in California suggested that LARC insertion skills were limited among PHC contraception providers, and about 60% of providers who formed part of the study saw the cost of instrumentation as a barrier.^24^

Practitioners’ views in terms of safety, efficacy and acceptability regarding LARC were studied in the general practice sector of the United Kingdom.^24^ Lack of skill in providing LARC was identified in 61% of participants as a barrier, and about 50% of participants believed that irregular bleeding deterred women from using LARC. Twenty percent were concerned about high discontinuation rates, and misconceptions about the side effects of contraceptive methods were common.^25^

A systematic review identified the following factors as barriers to the provision of LARC: (1) misconceptions regarding the risk of pelvic inflammatory disease (PID), (2) infertility and ectopic pregnancies, (3) misconceptions about the difficulty and risks of insertion of IUCDs, and (4) misconceptions about the mechanism of action. Health system barriers that were identified included confusing pharmaceutical guidelines, lack of understanding of the value and cost-effectiveness of LARC, and mandatory Pap smear screening for cervical cancer before insertion of an IUCD. Women’s lack of awareness and understanding of LARC, the fear of IUCDs and discontinuations because of bleeding pattern changes were identified as user barriers.^26^

In Malawi, a high workload, inconsistent supply of LARC and poor resources posed challenges to LARC provision. Providers had a favourable perception of LARC, but few felt competent to provide it.^27^ In Nigeria, more than 50% of providers perceived the provision of contraceptives for unmarried adolescents as promoting sexual promiscuity, and about 50% reported that unmarried adolescents should be asked to abstain from sex rather than providing them with contraceptives. Over a third reported that contraception services for both married and unmarried adolescents should not be provided.^28^ In Ghana, lack of IUCD-specific knowledge, provider discomfort with insertion and incomplete contraceptive counselling contribute to the lack of IUCD use.^29^ Research conducted in Egypt found that receiving formal contraception training was a significant predictor of recommending LARC and that providers generally had negative attitudes towards LARC prior to training.^30^

No previous study on LARC, with the aim of evaluating knowledge, beliefs and practices of nurse providers, has been performed in the Western Cape. The researcher was employed in KESS at the time of the study and identified a deficiency in LARC provision. This research aimed to evaluate the current knowledge, beliefs and practices of providers pertaining to the provision of LARC in the KESS, Western Cape, and to identify potential solutions to address barriers. The wider benefit could potentially prevent unwanted and unplanned pregnancies as well as the complications and sequelae associated with them.

## Research methods and design

### Study design

This quantitative study took the form of a descriptive survey and was conducted using a validated questionnaire.

### Setting

This study was conducted among nursing staff working in PHC centres in the public sector of the Eastern metropole of the Western Cape province in South Africa. This metropole has a population of 900 437 according to the 2011 census, with a headcount of two million patients over the age of five years who visit public sector PHC facilities annually, according to recent routine data collection at facilities. The Khayelitsha Eastern Substructure (KESS), a health substructure within the Eastern metropole, has been identified as the study population.

### Study population

Nursing staff who work in the dedicated women’s health section of the PHC facilities within KESS and who provide contraception were invited to participate in the study. Nursing staff from both the Department of Health (DOH) and City of Cape Town (COCT) PHC facilities were included.

Participants were selected by contacting the facility managers of each of the 29 PHC facilities in the KESS to identify nursing staff who provide contraception at the respective facilities.

Inclusion criteria were nurses who provide contraception in dedicated women’s health sections, employed within the KESS public sector and permanent nursing staff who have been employed for at least 1 year.

Agency, locum and community service nurses were excluded from the study. The locum group was excluded from the study because of unfamiliarity with the substructure or short periods of employment within the substructure, usually relieving permanent staff members during annual and sick leave or in the case of staff shortages.

### Data collection

A questionnaire from a previously conducted international study was validated and adapted to make the questionnaire more appropriate for use in the South African setting.^23^ The questionnaire on which the survey is based was developed in San Francisco in 2009. A United States national representative sample of nurse practitioners in primary care and women’s health were surveyed.^24^ The survey evaluated clinician characteristics, professional training, practice factors, the patient population and contraceptive care. Long-acting reversible contraceptive methods were evaluated by asking about clinician knowledge, insertion skills, perceptions of safety, patient eligibility, indications and need for training in IUCD and implant insertion. The adapted questionnaire used in this study evaluated the same components and circumstances around LARC insertion, with the addition of a section on common misconceptions.

The questionnaire used in this research was thus further validated and adapted by a panel of experts working with contraception. The panel consisted of two district gynaecologists, a gynaecologist working in tertiary care with a special interest in contraceptive care, four family physicians, a statistician and the study supervisor. Validation was acquired by sending the questionnaires to the relevant experts, incorporating their recommendations and sending them back to the experts after adjustments for final approval.

This questionnaire was piloted on five representatives of the study population from another substructure to address potential question ambiguity, logistics, feasibility and completion time. These subjects did not form part of the study population, and data obtained following the completion of the questionnaires were not included in the study. The feedback obtained after piloting aided to adjust the phrasing and style of questions and improved logistics and ambiguity.

Facility managers of each of the PHC facilities were contacted to arrange a suitable time for the completion of questionnaires. The staff involved in data collection were identified prior to the visit in discussion with each facility manager. Consent was discussed verbally with the participants, and a consent form provided for completion by the participants was signed prior to the completion of the questionnaire. The consent form explained how data would be kept anonymous and participants’ confidentiality maintained.

Questionnaires were administered by the researcher during a visit to the facility and were delivered face-to-face. The researcher was present during the completion. Completed questionnaires were collected immediately after completion. Face-to-face data collection was chosen to allow the participants to ask questions addressed to the researcher.

### Data analysis

The data were captured onto a spreadsheet and checked for any errors or omissions. Knowledge was tested by answering questions on insertion eligibility criteria. Likert scales were used for beliefs and misconceptions, and yes or no questions and numeric values were used for the sections on the practices of the participants. The data were then uploaded to the Statistical Package for the Social Sciences (SPSS) version 25 and analysed. Frequencies and percentages were used for categorical data. For numerical data, means and standard deviations or medians and interquartile range were used, depending on whether the variables were normally or not normally distributed. For inferential analysis, Pearson’s correlation tests and Pearson’s chi-square tests were used. Statistical significance was described as *p* < 0.05.

### Ethical considerations

Ethics approval was obtained from Stellenbosch University Health Research Ethics Committee (HREC) (Reference number: S18/10/204), and permission for data collection was obtained from the Western Cape Department of Health (DOH) and from the City of Cape Town (COCT). Participation was voluntary, anonymous and by means of informed consent.

## Results

### Profile of participants

The response rate was 80% (72 participants out of 90 nursing staff members who provide contraception in KESS PHC facilities). The remainder of staff members who met inclusion criteria were on leave (annual, maternity and sick leave), while the rest were on courses or at outreach facilities at the time of data collection. One out of the 29 facilities did not participate because a section of the facility had previously burnt down and nursing staff providing contraception had been relocated to nearby facilities. They were, however, included in the study, being employed at different facilities. Table 1 presents the demographic characteristics of the participants. The mean age of the participants was 41.3 years with s.d. 9.9, 95.8% were female and 41.7% obtained their highest qualification between 2001 and 2010.

**TABLE 1:** Respondent and practice characteristics (*N* = 72).

Characteristic	*N*	%
**Professional qualification**		
Advanced midwife	1	1.4
Clinical nurse practitioner	21	29.2
Professional nurse	41	56.9
Enrolled nurse	8	11.1
Enrolled nurse assistant	1	1.4
**Gender**		
Female	69	95.8
Male	3	4.2
**Period in which the highest qualification obtained**		
2011–2018	18	25.0
2001–2010	30	41.7
1996–2000	16	22.2
1988–1995	8	11.1
**Facility**		
COCT	53	73.6
DOH	19	26.4

COCT, City of Cape Town; DOH, Department of Health

**FIGURE 1:**
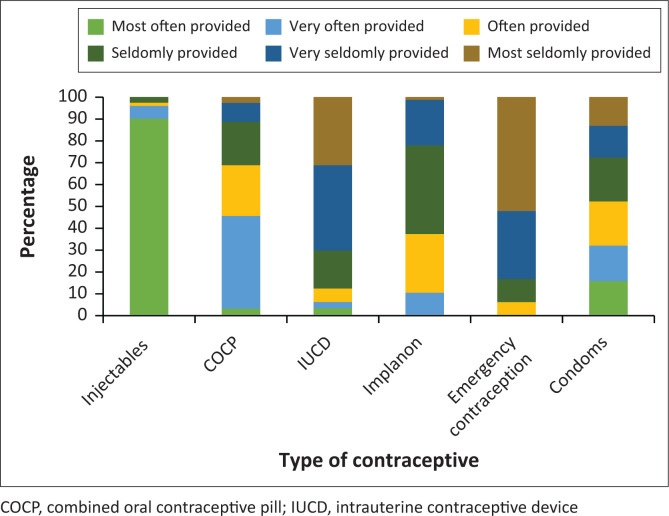
Types of contraception provided.

Figure 1 depicts the type of contraception provided. Only about 10% of participants provided the IUCD regularly, while the Implanon is provided regularly by about 40% of participants. Injectable contraception and the combined oral contraceptive pill (COCP) are noted to be provided most often in KESS facilities.

### Knowledge and training

The knowledge of participants was tested by asking questions based on eligibility for LARC used in South Africa and provided by the World Health Organization Medical Eligibility Criteria. It was shown that trained participants had superior knowledge about insertion criteria overall. A maximum score of 12/12 was awarded for IUCD and 11/11 for Implanon insertion eligibility criteria. The mean score for Implanon knowledge for those who were trained was 8.56 (s.d. 1.42) and for those who the untrained was 7.16 (s.d. 2.83) (*p* = 0.007). The mean score of participants trained in IUCD insertion was 10.42 (s.d. 1.80), and those who were untrained had a mean score of 8.03 (s.d. 3.70) (*p* = 0.019).

Trained staff also felt more confident inserting LARC than untrained staff, although only 61% of trained staff were confident in inserting IUCDs; 82% of trained staff were confident in the insertion of the Implanon. All untrained staff did not feel confident in the insertion of both methods of LARC. Staff who were both trained and felt confident in the insertion were low; 31.9% were trained and felt confident in IUCD insertion, while 56.1% were trained and felt confident in Implanon insertion.

In Table 2, information about training, insertion and confidence is shown. A lower proportion of staff trained to insert IUCDs performs actual insertions when compared to those trained for Implanon insertion, and confidence in IUCD insertion is low, while the Implanon group felt more confident in insertions. Nearly, all staff who are trained to insert the Implanon performed actual insertions. Overall, both the trained and untrained participants expressed a need for further training in the insertion of both methods. Poor knowledge and lack of training with poor confidence correlated with lower insertion rates.

**TABLE 2:** Training, insertion and confidence.

Level of training	IUCD insertion *n*†	IUCD insertion %†	Implanon insertion *n*‡	Implanon insertion %‡
Untrained	33	47.8	21	31.8
Trained	36	52.2	45	68.2
Trained and inserts	28	40.6	42	63.6
Trained and feels confident inserting	22	31.9	37	56.1
Needs further training	42	60.8	30	45.4

IUCD, intra-uterine contraceptive device

† , *N* = 69 for IUCD;

‡ , *N* = 66 for Implanon.

### Beliefs

Participants’ beliefs were evaluated with the use of Likert scales. Certain false beliefs and misconceptions would deter the provider from inserting LARC, while positive beliefs and attitudes towards LARC will motivate the provider to insert or refer to a trained provider who performs insertions.

Table 3 shows beliefs against a number of participants who are of the opinion that said belief is correct or incorrect. Most participants agreed with the true beliefs and disagreed with the false beliefs, this irrespective of their training or qualification. Common misconceptions were included. In the South African setting, there is a misconception that Implanons may be forcefully removed to be smoked as an illicit drug has, this has also been included in the questionnaire; 14% of providers believed that the IUCD can migrate, while 24% believed that the Implanon can migrate. Nearly, a quarter believed that Implanon can delay return in fertility. Misconceptions may be deterrents to both the provider and the user. Questions were asked on beliefs with regard to barriers to effective provision. Some of the systemic barriers to provision were time constraints, shortage of staff, privacy, stock and equipment, and inaccessibility to training courses.

**TABLE 3:** Beliefs regarding long-acting reversible contraceptives insertion (*N* = 72).

Beliefs regarding LARC	Yes		No		Unsure or no answer	
	*n*	%	*n*	%	*n*	%
**True beliefs**						
IUCD are safe	66	91.7	0	-	6	8.3
IUCD can be inserted immediately postpartum	51	70.9	16	22.2	5	6.9
IUCD can be inserted immediately post-abortion	55	76.4	7	9.7	10	13.9
Follow-up after IUCD insertion is necessary	69	95.8	0	-	3	4.2
Remove IUCD to treat PID	32	44.4	23	31.9	17	23.7
Implanon is safe	69	95.8	0	-	3	4.2
Implanon can be inserted immediately postpartum	68	94.4	3	4.2	1	1.4
Implanon can be inserted immediately post-abortion	69	95.8	1	1.4	2	2.8
Implanon can be inserted any time during menstrual cycle	58	80.6	6	8.3	8	11.1
**False beliefs and misconceptions**						
IUCD increases the risk of miscarriages	8	11.1	45	62.5	19	26.4
Association between IUCD and ectopic pregnancies	13	18.1	27	37.5	32	44.4
Association between IUCD and PID	16	22.2	35	48.6	21	29.2
IUCD can delay return in fertility	3	4.2	61	84.7	8	11.1
IUCD can migrate	10	13.9	44	61.1	18	25.0
Implanon can migrate	15	20.8	51	70.8	6	8.4
Implanon can be removed and smoked	8	11.1	44	61.1	20	27.8
Implanon can delay return in fertility	17	23.6	49	68.0	6	8.4
**Beliefs regarding barriers**						
Enough time to insert LARC	43	59.7	14	19.5	15	20.8
Enough space to insert LARC	48	66.7	8	11.1	16	22.2
Enough privacy to insert LARC	53	73.6	9	12.5	10	13.9
Always stock available	46	63.8	14	19.5	12	16.7
Training courses in LARC insertion easily accessible	40	55.5	21	29.2	11	15.3
Enough staff at facility	35	48.6	25	34.7	12	16.7
Correct equipment for insertion available	46	63.8	13	18.1	13	18.1
Another skilled person available for assistance	40	55.6	17	23.6	15	20.8
Guidelines on LARC insertion available	52	72.2	15	20.8	5	7.0

LARC, long-acting reversible contraceptives; IUCD, intrauterine contraceptive device; PID, pelvic inflammatory disease.

Table 4 demonstrates whether there is a correlation between years of experience and whether participants agreed with misconceptions by using Pearson’s correlation test. No correlation was found between participants who had more than 5 years of experience and whether they agreed with misconceptions, compared to participants with less than 5 years of experience. Correlation is significant at the 0.05 level (2-tailed). From these data, junior and senior staff shared similar beliefs with regard to misconceptions, and seniority is not a factor that would deter or encourage staff to provide LARC based on their beliefs about misconceptions.

**TABLE 4:** Correlating years (more than five years) of experience to common misconceptions.

Variable	IUCD delays fertility	IUCD can migrate	Implanon can migrate	Implanon can be smoked	Implanon delays fertility
Pearson’s correlation (r)	0.083	−0.010	−0.105	0.003	0.113
*p*-value	0.503	0.0933	0.389	0.981	0.354
*N*	68	67	69	68	69

IUCD, intrauterine contraceptive device

### Practices

The total number of patients counselled for contraception per week (7 days) prior to the study was 2759. Of those, only 40.1% (*n* = 1105) were counselled for IUCD, and 47.2% (*n* = 1303) were counselled for Implanon insertion. Participants who had more experience in years (> 5 years) did not counsel more patients (*p* > 0.01) and did not do more LARC insertion than those with less experience in years. Trained participants counselled more patients than untrained participants. While 72% of staff had evidence-based guidelines on LARC provision available, 65% used the guidelines.

Table 5 shows that in the IUCD insertion group, more participants from the two largest professional qualification groups (clinical nurse practitioner and professional nurse) did not insert IUCDs than those who did. In the Implanon group, more participants from the clinical nurse practitioner (CNP) and professional nurse (PN) groups performed insertions than those who did not. Using the chi-square test, one could infer that there was a statistically significant difference for providers who inserted Implanons in the larger qualification groups compared to the smaller qualification groups, while there was no statistically significant difference for IUCD insertion within these groups. Clinical nurse practitioners, advanced midwives (AMs) and professional nurses inserted more LARC than the enrolled nurses (ENs) and enrolled nursing assistants (ENAs). Staff with higher professional qualification insert more LARC than the less-qualified groups.

**TABLE 5:** Relationship between nursing qualification and practice.

Professional qualification	Inserts IUCD †*		Inserts Implanon ‡*	
	Yes	No	Yes	No
**Clinical nurse practitioner**				
*n*	7	14	12	9
%within PQ	33.3	66.7	57.1	42.9
%within insertion	25.0	31.8	28.6	32.1
**Advanced midwife**				
*n*	1	0	1	0
%within PQ	100	0	100	0
%within insertion	3.6	0	2.4	0
**Professional nurse**				
*n*	19	22	29	12
%within PQ	46.3	53.7	70.7	29.3
%within insertion	67.9	50.0	69.0	42.9
**Enrolled nurse**				
*n*	1	7	0	7
%within PQ	12.5	87.5	0	100
%within insertion	3.6	15.9	0	25
**Enrolled nursing assistant**				
*n*	0	1	-	-
%within PQ	0	100	-	-
%within insertion	0	2.3	-	-

IUCD, intrauterine contraceptive device; PQ, professional qualifications

† , χ² for IUCD insertion = 0.167;

‡ , χ² for Implanon insertion = 0.004.

* , *p*-value = 0.05.

## Discussion

The findings of this study provided a comprehensive evaluation of knowledge, beliefs and practices pertaining to LARC provision, and barriers specific to healthcare systems in KESS were identified. Most participants had more than 5 years’ experience and were able to provide an accurate account of practices. Of the participants who formed part of the study, most were PNs. This is likely because CNPs have a higher qualification and can manage a wider range of patients and diseases. They are usually not allocated to work in only one dedicated area.^31^ It was found that PNs had a higher percentage of staff trained for the insertion of both methods of LARC.

Similar to previous international studies and a South African household survey, participants in KESS reported that LARC were not as readily provided, and short-acting methods were mostly prescribed and/or administered.^1,2,3,4,5,6^

Counselling on LARC was poor among participants, and participants indicated that they counselled less than 50% of patients on LARC methods: 40.1% for IUCD and 47.2% for Implanon insertion. These findings correlated with a lack of knowledge and training on counselling on LARC methods and are comparable to research among PHC and specialised contraception provider nurses in the United States. PHC contraception providers only routinely counselled about half of patients on LARC methods.^32^ In Rwanda, government clinic nurses who provided contraception were studied. They indicated that those who were not trained to insert LARC or did not have time to provide them were reluctant to promote LARC.^33^ The findings are also in keeping with other research undertaken in Southern Africa, suggesting an unmet need at the provider level for training to competency in LARC methods, appropriate counselling, and insertion and removal techniques.^34^ South African providers felt that they had limited knowledge and had the need for further training in counselling and insertion of LARC.^35^

Common misconceptions were evaluated. There was no difference between more and less experienced participants and whether they were of the opinion that the common misconceptions were true. To date, no previous literature had been found that studied these relationships.

Participants had a favourable perception of LARC methods regarding safety. Timing of insertion of both devices and the importance of follow-up were well understood by participants. Beliefs with regard to associations between LARC methods and conditions such as miscarriages, ectopic pregnancies and PID varied among participants of all qualification groups. There was no correlation between years of practice, professional qualification and beliefs regarding LARC methods. These findings were similar among those trained and untrained for LARC provision. A 2012 study in the United States evaluated beliefs and perceptions of PHC contraception providers and results yielded similar findings to those studies in this research. Over half of the participants from the United States study thought that LARC methods were safe and suitable for a wide variety of women. The study was conducted in 2012 after LARC gained more popularity and training courses became more readily available to staff.^36^

The findings of this study show that a lack of training is associated with poorer confidence and poorer knowledge on eligibility criteria for insertion. Trained participants counselled more patients on LARC methods and performed more insertions. They also had superior knowledge on insertion criteria. These findings are in keeping with studies conducted in first-world countries such as the United States and the United Kingdom, which found that superior knowledge on LARC eligibility criteria, counselling, and insertion skills could be attributed to training.^19,20,21,37^ These findings were also observed in the African context with research done in Egypt.^29^

Lower provision of LARC had an association with poor or inaccurate knowledge and insufficient numbers trained for insertion. These findings are comparable to an Australian systematic review suggesting that IUCD training for healthcare providers contributes to improved knowledge and attitudes regarding the provision of IUCDs, high rates of successful insertions with low complication rates and increased provision of IUCDs to women.^38^

Lack of resources such as time constraints, limited stock and equipment, and the absence of another skilled person at the facility were suggested as barriers to LARC insertion by participants in this study (21%, 20% and 25%, respectively). Over a third of participants believed that a shortage of staff was a barrier and almost a third thought that training courses were not easily accessible. There are limited studies available on healthcare and system barriers; however, an international literature review and a survey among LARC experts found that lack of skilled providers onsite, lack of trained contraception providers, and cost and shortages of instrumentation are barriers to LARC provision.^26,39^ Two African studies conducted in Malawi and Uganda found that systemic problems such as lack of experienced LARC providers, high work burden and lack of equipment have resulted in reliance on outside referrals for LARC insertion.^27,40^

### Strengths and limitations

All facilities in the Khayelitsha Eastern Substructure were included in the study, and there was a good response rate for the completion of questionnaires. The data collection tool had been validated and may therefore be used in future research.

Questionnaires were mostly completed by staff who provided contraception. However, two facility managers from the 28 facilities that participated asked all the nursing staff employed at their facilities to complete the questionnaires. During the completion, it was clear that not all of them provided contraception regularly and may not have understood all questions, specifically questions pertaining to knowledge. However, it is unlikely that this would have affected the results of the study significantly. The analysis could have been taken further in terms of the direct correlation of factors with LARC provision. This is an opportunity for further research.

### Recommendations

From the above findings, recommendations can be made to improve LARC provision. Hands-on training, regular refresher courses and a trained mentor or supervisor at each facility may improve provision, together with supporting contraception providers to attend courses. Dedicated LARC providers may improve uptake by women. Community health workers may also have a role in increasing uptake by doing initial counselling and expanding women’s knowledge on different methods of contraception. Long-acting reversible contraceptives may be offered to adolescents and first-time contraception users. Policymakers should ensure that an allocated space for women’s health and contraception provision is available at facilities that would address space and privacy constraints. Compulsory courses on counselling should be made available for all healthcare workers, even those who do not provide contraception services, and education on LARC methods should form part of teaching in schools to improve awareness among adolescents. In the future, perspectives from the users should be researched to identify factors that will improve the uptake of LARC.

## Conclusion

The knowledge, beliefs and practices pertaining to LARC provision differ among healthcare providers with different levels of training and qualification. Poor knowledge was identified in the untrained providers and in the groups with lower levels of qualification. Many participants indicated a further need for training in LARC insertion. Provision of LARC, particularly the IUCD, was among the lowest of contraception provided, and lack of knowledge and poor confidence attributed to the lower figures of LARC insertion. Participants had a favourable perception regarding the safety of LARC methods, and there was no correlation between more years of experience, counselling and beliefs. To date, no comparable studies were found pertaining to these relationships. Poor knowledge regarding insertion criteria, poor confidence in LARC insertion and inadequate numbers of women counselled were identified as the major factors that limit the provision of LARC, and all had a relationship with lack of training. Specific system barriers have been identified that may be addressed to improve LARC uptake. The evaluation in this study is in keeping with previous findings in terms of knowledge, beliefs and practices regarding the provision of LARC.

## References

[1] Chersich MF, Wabiri N, Risher K, et al. Contraception coverage and methods used among women in South Africa: A national household survey. S Afr Med J 2017;107(4):307–314. 10.7196/samj.2017.v107i4.12141 28395681

[2] Olson EM, Kramer RD, Gibson C, Wautlet K, Schmuhl NB, Ehrenthal D. Health care barriers to provision of long-acting reversible contraception in Wisconsin. Wis Med J 2018;117(4):149–155. PMC6734562 30407764

[3] Joshi R, Khadilkar S, Patel M. Global trends in use of long-acting reversible and permanent methods of contraception: Seeking a balance. Int J Gynecol Obstet 2015;131:S60–S63. 10.1016/j.ijgo.2015.04.024 26433510

[4] Phillips J, Sandhu P. Barriers to implementation of long-acting reversible contraception: A systematic review. J Am Assoc Nurse Pract 2018;30(4):236–243. 10.1097/JXX.0000000000000019 29757790

[5] Neukom J, Chilambwe J, Mkandawire J, Mbewe RK, Hubacher D. Dedicated providers of long-acting reversible contraception: New approach in Zambia. Contraception 2011;83(5):447–452. 10.1016/j.contraception.2010.08.021 21477688

[6] Adeyemi AS, Adekanle DA, Komolafe JO. Pattern of contraceptives choice among the married women attending the family planning clinic of a tertiary health institution. Niger J Med 2008;17(1):67–70. 10.4314/njm.v17i1.37359 18390137

[7] Lotke PS. Increasing use of long-acting reversible contraception to decrease unplanned pregnancy. Obstet Gynecol Clin North Am 2015;42(4):557–567. 10.1016/j.ogc.2015.07.008 26598299

[8] Builu PM, Naidoo TD. Attitudes towards and knowledge about intrauterine contraceptive devices among women in the reproductive age group in a resource-constrained setting. S Afr J Obstet Gynaecol 2015;21(2):27–32. 10.7196/sajog.950

[9] Rees H, Pillay Y, Mullick S, Chersich MF. Strengthening implant provision and acceptance in South Africa with the ‘any woman, any place, any time’ approach: An essential step towards reducing unintended pregnancies. S Afr Med J 2017;107(11):939–944. 10.7196/SAMJ.2017.v107i11.12903 29400025

[10] Sedgh G, Singh S, Hussain R. Intended and unintended pregnancies worldwide in 2012 and recent trends. Stud Fam Plann 2014;45(3):301–314. 10.1111/j.1728-4465.2014.00393 25207494 PMC4727534

[11] Black AY, Guilbert E, Hassan F, et al. The cost of unintended pregnancies in Canada: Estimating direct cost, role of imperfect adherence, and the potential impact of increased use of long-acting reversible contraceptives. J Obstet Gynaecol Can 2015;37(12):1086–1097. 10.1016/S1701-2163(16)30074-3 26637081 PMC5527291

[12] Parks C, Peipert JF. Eliminating health disparities in unintended pregnancy with long-acting reversible contraception (LARC) Presented in part at the 2013 annual meeting of the American Gynecological and Obstetrical Society. Am J Obstet Gynecol 2016;214(6):681–688. 10.1016/j.ajog.2016.02.017 26875950 PMC4884485

[13] Asekun-Olarinmoye EO, Adebimpe WO, Bamidele JO, Odu OO, Asekun-Olarinmoye IO, Ojofeitimi EO. Barriers to use of modern contraceptives among women in an inner city area of Osogbo metropolis, Osun State, Nigeria. Int J Women’s Health 2013;5:647–655. 10.2147/IJWH.S47604 24143124 PMC3797631

[14] WHO. Medical eligibility criteria for contraceptive use. 5th ed. Geneva: World Health Organization; 2015. 26447268

[15] Birgisson NE, Zhao Q, Secura GM, Madden T, Peipert JF. Preventing unintended pregnancy:The contraceptive CHOICE project in review. J Women’s Health 2015;24(5):349–353. http://doi-org.ez.sun.ac.za/10.1089/jwh.2015.5194 10.1089/jwh.2015.5191 PMC4441000 25825986

[16] Harper CC, Rocca CH, Thompson KM, et al. Reductions in pregnancy rates in the USA with long-acting reversible contraception: A cluster randomised trial. Lancet 2015;386(9993):562–568. 10.1016/S0140-6736(14)62460-0 26091743 PMC12987576

[17] Stoddard A, McNicholas C, Peipert JF. Efficacy and safety of long-acting reversible contraception. Drugs 2011;71(8):969–980. 10.2165/11591290-000000000-00000 21668037 PMC3662967

[18] Shoupe D. LARC methods: Entering a new age of contraception and reproductive health. Contracept Reprod Med 2016;1(1):1–9. 10.1186/s40834-016-0011-8 29201394 PMC5675060

[19] Speidel JJ, Harper CC, Shields WC. The potential of long-acting reversible contraception to decrease unintended pregnancy. Contraception 2008;78(3):197–200. 10.1016/j.contraception.2008.06.001 18692608

[20] Rubin SE, Davis K, McKee MD. New York city physicians’ views of providing long-acting reversible contraception to adolescents. Ann Fam Med 2013;11(2):130–136. 10.1370/afm.1450 23508599 PMC3601390

[21] Pritt NM, Norris AH, Berlan ED. Barriers and facilitators to adolescents’ Use of long-acting reversible contraceptives. J Pediatr Adolesc Gynecol 2017;30(1):18–22. 10.1016/j.jpag.2016.07.002 27477904

[22] Hoggart L, Walker S, Newton VL, Parker M. Provider-based barriers to provision of intrauterine contraception in general practice. BMJ Sex Reprod Health 2018;44(2): 82–89. 10.1136/bmjsrh-2017-101798 29921629

[23] Gomez AM, Fuentes L, Allina A. Women or LARC first? Reproductive autonomy and the promotion of Long-Acting Reversible Contraceptive methods. Perspect Sex Reprod Health 2014;46(3):171–175. 10.1363/46e1614 24861029 PMC4167937

[24] Biggs MA, Harper CC, Malvin J, Brindis CD. Factors influencing the provision of long-acting reversible contraception in California. Obstet Gynecol. 2014;123(3):593–602. 10.1097/AOG.0000000000000137 24499746

[25] Wellings K, Zhihong Z, Krentel A, Barrett G, Glasier A. Attitudes towards long-acting reversible methods of contraception in general practice in the UK. Contraception. 2007;76(3):208–242. 10.1016/j.contraception.2007.05.085 17707718

[26] Black K, Lotke P, Buhling KJ, Zite NB and on behalf of the Intrauterine contraception for Nulliparous women: Translating Research into Action (INTRA) group. A review of barriers and myths preventing the more widespread use of intrauterine contraception in nulliparous women. Eur J Contracept Reprod Health Care 2012;17(5):340–348. 10.3109/13625187.2012.700744 22834648 PMC4950459

[27] Mwafulirwa T, Shea MSO, Hamela G, et al. Family planning providers’ experiences and perceptions of Long - Acting Reversible Contraception in Lilongwe, Malawi. Contraception 2014:90(3):62–71. 10.1016/j.contraception.2014.05.070 29553165

[28] Ahanonu EL. Attitudes of healthcare providers towards providing contraceptives for unmarried adolescents in Ibadan, Nigeria. J Family Reprod Health 2014;8(1):33–40. 24971131 PMC4064762

[29] Robinson N, Moshabela M, Owusu-Ansah L, Kapungu C, Geller S. Barriers to intrauterine device uptake in a rural setting in Ghana. Health Care Women Int 2016;37(2):197–215. 10.1080/07399332.2014.946511 25153448 PMC4450130

[30] Aziz M, Ahmed S, Ahmed B. Attitudes of physicians providing family planning services in Egypt about recommending intrauterine device for family planning clients. Sex Reprod Healthcare 2017;14:64–68. 10.1016/j.srhc.2017.09.004 29195636

[31] Kapp R, Mash RJ. Perceptions of the role of the clinical nurse practitioner in the Cape Metropolitan doctor-driven community health centres. S Afr Fam Pract 2004;46(10):21–25. 10.1080/20786204.2004.10873150

[32] Ryan E. Contraceptive practices of advanced practice registered nurses in outpatient settings. University of North Carolina; 2017.

[33] Mazzei A, Ingabire R, Mukamuyango J, et al. Community health worker promotions increase uptake of long-acting reversible contraception in Rwanda. Reprod Health. 2019;16(1):1–11. 10.1186/s12978-019-0739-0 31164155 PMC6549304

[34] Morse J, Chipato T, Blanchard K, et al. Provision of long-acting reversible contraception in HIV-prevalent countries: Results from nationally representative surveys in Southern Africa. BJOG: Int J Obstet Gynaecol 2013;120(11):1386–1393. 10.1111/1471-1528.12290 PMC3775997 23721413

[35] Gutin SA, Mlobeli R, Moss M, Buga G, Morroni C. Survey of knowledge, attitudes and practices surrounding the intrauterine device in South Africa. Contraception 2011;83(2):145–150. 10.1016/j.contraception.2010.07.009 21237340

[36] Philliber AE, Hirsch H, Brindis CD, Turner R, Philliber S. The use of ACOG guidelines: Perceived contraindications to IUD and implant use among family planning providers. Matern Child Health J 2017;21(9):1706–1712. 10.1007/s10995-017-2320-1 28707101

[37] Thompson KMJ, Rocca CH, Stern L, et al. Training contraceptive providers to offer intrauterine devices and implants in contraceptive care: A cluster randomized trial. Am J Obstet Gynecol 2018;218(6):597. 10.1016/j.ajog.2018.03.016 PMC5970088 29577915

[38] Ouyang M, Peng K, Botfield JR, McGeechan K. Intrauterine contraceptive device training and outcomes for healthcare providers in developed countries: A systematic review. PLoS One 2019;14(7):1–14. 10.1371/journal.pone.0219746 PMC6629157 31306443

[39] Foster DG, Barar R, Gould H, Gomez I, Nguyen D, Biggs MA. Projections and opinions from 100 experts in long-acting reversible contraception. Contraception 2015;92(6):543–552. 10.1016/j.contraception.2015.10.003 26515195

[40] Mugisha JF, Reynolds H. Provider perspectives on barriers to family planning quality in Uganda : A qualitative study. J Fam Plann Reprod Health Care 2008;34(1):37–41. 10.1783/147118908783332230 18201405

